# Depression and Suicide Risk Among Nursing Staff at a Honduran National Hospital

**DOI:** 10.7759/cureus.45520

**Published:** 2023-09-19

**Authors:** Andrea P Rodríguez Rodríguez, Eliezer D Acosta Romero, Luis S Jananía Gámez, Juan F Ortiz Mairena, Carlos E Meraz Cardona, Carlos O Hernández Granados, Reenie H Pineda Villeda

**Affiliations:** 1 Paediatrics, Mario Catarino Rivas Hospital, San Pedro Sula, HND; 2 Internal Medicine, Mario Catarino Rivas Hospital, San Pedro Sula, HND; 3 General Surgery, Mario Catarino Rivas Hospital, San Pedro Sula, HND

**Keywords:** beck depression inventory-ii, okasha assessment tool, honduran national hospital, nursing staff, suicide risk, depression

## Abstract

Background: Depression and suicide risk among nursing staff have become increasingly concerning, especially given the demanding nature of their profession. The World Health Organization identifies depression as a primary factor contributing to global disability and suicide deaths.

Methods: A descriptive, non-experimental, cross-sectional cohort study was conducted, encompassing the eligible personnel (n=82) out of a total of 102 nurses at the Mario Catarino Rivas Hospital in San Pedro Sula, Honduras, from October to November 2022. The study utilized the Okasha assessment tool to gauge the prevalence of suicidal risk and the Beck Depression Inventory-II (BDI-II) instrument to analyze the extent and severity of depression. In addition, the participants completed a demographic survey.

Results: The average age of participants was 34.91 years, with a majority (86.6%) being female. In terms of work assignments, 54.9% were employed in the inpatient area. Regarding the mental health of the nursing staff, 78% displayed no or minimal depression, 9.7% presented mild depression, 7.3% showed moderate depression, 4.8% displayed severe depression, and 14.6% exhibited a suicide risk. Young adults had the highest prevalence of all three levels of depression, and the emergency department and inpatient area had the most at-risk individuals for suicidal tendencies.

Conclusion: The study offers a comprehensive insight into the demographics, work environment, and mental health of the nursing staff at the Honduran National Hospital. The results highlight the importance of specialized measures and strong support systems to safeguard the mental health of nursing staff.

## Introduction

The World Health Organization has classified depression as the leading contributor to global disability and emphasizes that depression is the primary factor contributing to suicide deaths [1]. There is a total of 322 million people worldwide with depression and approximately 703,000 individuals die by suicide [2].

In the Americas, suicide ranks as the third leading cause of death for those aged 20-24 years, with the highest suicide rate observed among individuals aged 45-59. Between 2015 and 2019, around 98,000 suicides were reported annually, with a higher non-Hispanic suicide rate in North America and the Caribbean compared to the regional rate [3].

In 2016, the most recent Honduran statistics revealed that 55.7% of adults exhibited symptoms suggesting the presence of a mental disorder, with a depression prevalence of 30.7% among them. The coronavirus disease 2019 (COVID-19) pandemic further exacerbated the country's overall situation [3].

Depression and suicide risk among nursing staff is a reality; healthcare personnel face long work hours, job-related stress, and overwhelming emotional or physical demands [4]. Tools such as the Okasha assessment tool and the Beck Depression Inventory-II (BDI-II) instrument can analyze suicide risk and depression [5, 6].

The existing literature provides extensive data on the prevalence of depression and suicide risk among healthcare professionals globally. However, there is a noticeable lack of focused studies that describe the relationship between work environment, age, and mental health outcomes among nursing staff. This study aims to fill these gaps by providing a comprehensive report of the prevalence and severity of depression and suicide risk among nursing staff at a Honduran National Hospital.

The rationale for this study stems from the need to understand the mental health challenges faced by nursing staff in a specific healthcare setting in Honduras, a country with unique healthcare challenges including limited resources and high stress. Moreover, this study aims to go beyond general statistics by segmenting data based on age and work department to identify high-risk groups within the nursing staff, thereby providing the groundwork for more targeted mental health interventions.

## Materials and methods

We devised and executed a descriptive, non-experimental, cross-sectional cohort study that incorporated both quantitative and qualitative elements. In this study, we employed a convenience sampling technique and the study population comprised the nursing personnel that fit the inclusion criteria (n=82) out of a total population of 102 who were employed in various departments at the Mario Catarino Rivas Hospital, the largest national hospital in the northern region of the country of Honduras, Central America located in the city of San Pedro Sula. The participants were individuals who satisfied the predefined inclusion criteria: adult members (above age 18) of the nursing staff working in Mario Catarino Rivas Hospital willing to participate in the investigation. The study was conducted over two months from October 1, 2022, to November 30, 2022. The study excluded all individuals who did not belong to the nursing staff at the hospital. The data collection instruments were the Okasha assessment tool and the BDI-II. 

The objectives of this study were as follows: firstly, to develop a comprehensive profile of the nursing personnel employed at the Honduran National Hospital; secondly, to identify the age bracket that exhibits depression and suicide risk; thirdly, to determine the specific hospital units with depression and suicide risk; fourthly, to find the prevalence of suicidal risk within the nursing staff through the utilization of the Okasha assessment tool; and finally, to analyze the severity of depression among the nursing staff by employing the BDI-II instrument.

The Okasha assessment tool, a self-report questionnaire administered in paper-based format, is designed to gauge the risk of suicide. This tool comprises questions related to suicidal ideation and suicide attempts. The scoring involves summing the first three questions to form a sub-score for suicidal ideation, which ranges from 0 to 9 points. This sub-score is then added to the score for the question about suicide attempts, resulting in a total suicidality score that can range from 0 to 12 points. A score of 5 or higher serves as the cutoff for increased suicide risk. The second instrument was the BDI-II, also a self-report, paper-based inventory consisting of 21 multiple-choice questions related to symptoms of depression. Each question is scored from 0 to 3, and the total score can range from 0 to 63, with higher scores indicating more severe depressive symptoms [5,6]. Both instruments were anonymously administered in Spanish to suit the Honduran context and have well-established reliability and validity. In addition to these instruments, the participants were asked to anonymously complete a paper-based survey administered in Spanish to describe demographic characteristics.

After obtaining official approval from the medical director and director of human resources of the Mario Catarino Rivas Hospital, and the National Autonomous University of Honduras ethics committee with approval code 1696-2022, we proceeded to collect the necessary data for our study based on one-on-one surveys. Subsequently, the collected data underwent comprehensive processing using the IBM SPSS Statistics for Windows, Version 29.0 (Released 2022; IBM Corp., Armonk, New York, United States).

## Results

**Figure 1:**
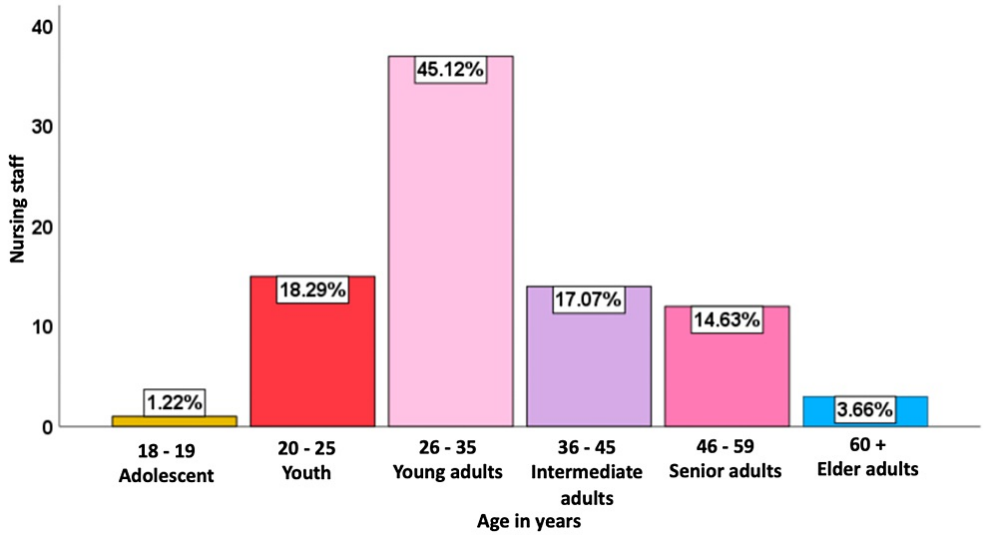
Age distribution among participants (n=82)

Out of the 102 eligible nursing staff, 82 participants responded to the survey. Among the participants, the average age was 34.91 years with 45.1% (n=37) falling within the 26-35 age range (Young adults), 18.3% (n=15) between 20 and 25 years (Youth), 17% (n=14) belonging to the 36-45 age group (Intermediate adults), 14.6% (n=12) aged 46-59 (Senior adults), 3.7% (n=3) were 60 years or older (Elder adults), and 1.2% (n=1) were 18 or 19 years old (Adolescents) (Figure 1); 86.6% (n=71) were female and 13.4% (n=11) were male. In relation to marital status, 51.2% (n=42) of participants were single, 23.2% (n=19) were married, 18.3% (n=15) were civil partners, 4.9% (n=4) were divorced, and 2.4% (n=2) were widowed.

Among the participants, 13.41% (n=11) had both hypertension and type 2 diabetes (DM2), 9.76% (n=8) had DM2 only, 10.98% (n=9) had hypertension only, 2.44% (n=2) had other medical conditions, while 63.41% (n=52) had no medical conditions. Out of the individuals with medical conditions, 80% (n=24) are utilising medications as part of their treatment regimen, while the remaining 20% (n=6) rely solely on lifestyle modifications.

When it came to their drinking habits, a majority, 53.66% (n=44), stated that they did not consume any alcohol, 30.49% (n=25) reported drinking one to three units, and 15.85% (n=13) indicated consuming four to six units of alcohol per week. Out of the 38 who drank alcohol, 10.52% (n=4) reported drinking during weekdays while 89.47% (n=34) drank during weekends.

In relation to their smoking habits, the majority, 93.90% (n=77), were non-smokers, while a minority, 6.10% (n=5), reported smoking, of whom 60% (n=3) had smoked for more than five years while 40% (n=2) had smoked for less than five years.

In terms of work assignments, 54.9% (n=45) were employed in the inpatient area, 35.4% (n=29) worked in the emergency department, 8.5% (n=7) worked in outpatient consultation, and 1.2% (n=1) worked in administrative roles. Furthermore, 15.9% (n=13) of participants worked in more than one institution, while 84.2% (n=69) exclusively worked in a single institution.

Regarding the COVID-19 pandemic, 76.8% (n=63) worked during it while 23.2% (n=19) did not. Among the 19 individuals who were not employed during the pandemic, 89.5% (n=17) requested leave due to preexisting medical conditions while 10.52% (n=2) were directly affected by the virus. In terms of work schedule flexibility, 78% (n=64) had a modifiable work schedule according to the legal contract allowing them to switch shifts and change the amount of hours worked after discussion with management. In comparison, 22% (n=18) did not have this flexibility.

The distribution of participants' years of employment at the hospital was as follows: 31.7% (n=26) had worked for one to five years, 25.6% (n=21) had worked for less than one year, 20.7% (n=17) had worked for 6-10 years, 7.3% (n=6) had worked for 11-15 years, 14.6% (n=12) had worked for more than 15 years at the hospital. In terms of daily work hours, the breakdown of participants was as follows: 67.1% (n=55) worked eight hours a day, 3.7% (n=3) worked less than eight hours, and 29.3% (n=24) worked more than eight hours.

Regarding the participants' mental health status, 78% (n=64) were classified as having either no or minimal depression, 9.8% (n=8) with mild depression, 7.3% (n=6) with moderate depression, and 4.9% (n=4) with severe depression. Regarding suicide risk, 14.6% (n=12) exhibited such risk, while 85.4% (n=70) did not.

Among the young adults, 35.4% (n=29) had no or minimal depression, 3.7% (n=3) showed moderate and mild depression showed each, and 2.4% (n=2) exhibited severe depression. In the youth group, the study found 11% (n=9) with no or minimal depression, and severe depression, moderate and mild depression were shown in 2.4% each (n=2). For intermediate adults, 14.6% (n=12) had no or minimal depression while 2.4% (n=2) experienced mild depression. Adolescents accounted for 1.2% (n=1) with moderate depression. Among senior citizens, 2.4% (n=2) had no or minimal depression and 1.2% (n=1) had mild depression. Lastly, among elder adults, 14.6% (n=12) had no or minimal depression.

**Figure 2:**
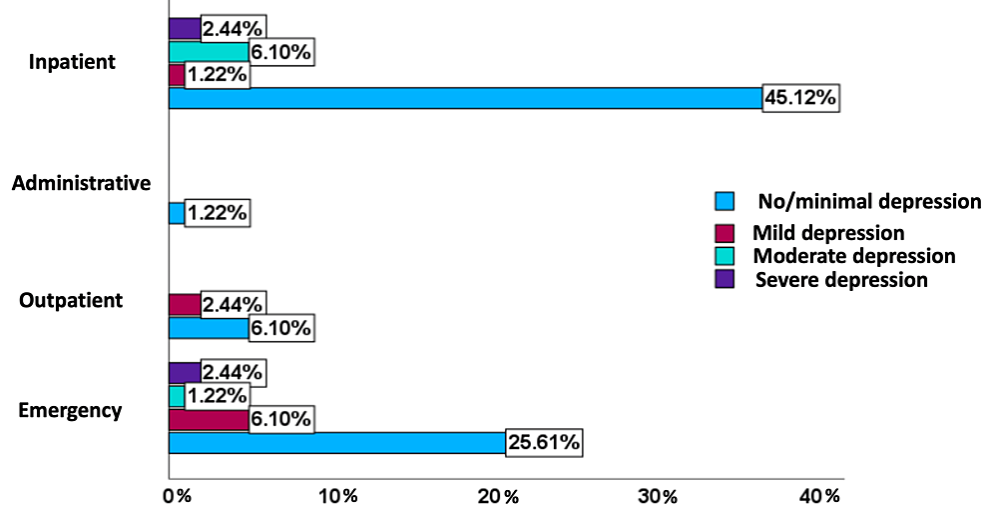
Distribution of severity of depression based on nursing staff's work area in a Honduras National Hospital

The severity of depression was compared to the area of work (Figure 2). In the inpatient area, 45.1% (n=37) had no or minimal depression, 1.2% (n=1) experienced mild depression, 6.1% (n=5) showed moderate depression and 2.4% (n=2) of the nursing staff exhibited severe depression. Within the emergency department, 25.6% (n=21) had no or minimal depression, 6.1% (n=5) displayed mild depression, 1.2% (n=1) demonstrated moderate depression, and 2.4% (n=2) had severe depression. In the outpatient consultation area, 6.1% (n=5) showed no or minimal depression, and 2.4% (n=2) had mild depression. For the administrative area, 1.2% (n=1) have no depression.

From the 12 nurses who presented a suicidal risk, distribution varied among different age groups: young adults accounted for 50% (n=6), youth for 25% (n=3), senior citizens for 8.3% (n=1), intermediate adults for 8.3% (n=1), and adolescents for 8.3% (n=1). Out of the patients presenting suicide risk, 50% (n=6) belonged to the emergency department and 41.7% (n=5) worked in the inpatient department.

Among the 29 participants employed in the emergency department, 21% (n=6) exhibited a risk of suicide; within the inpatient sector, 11.1% (n=5) out of 45 participants showed a risk of suicide; and in the outpatient consultation area, 1.4% (n=1) out of seven participants displayed a risk of suicide.

**Figure 3:**
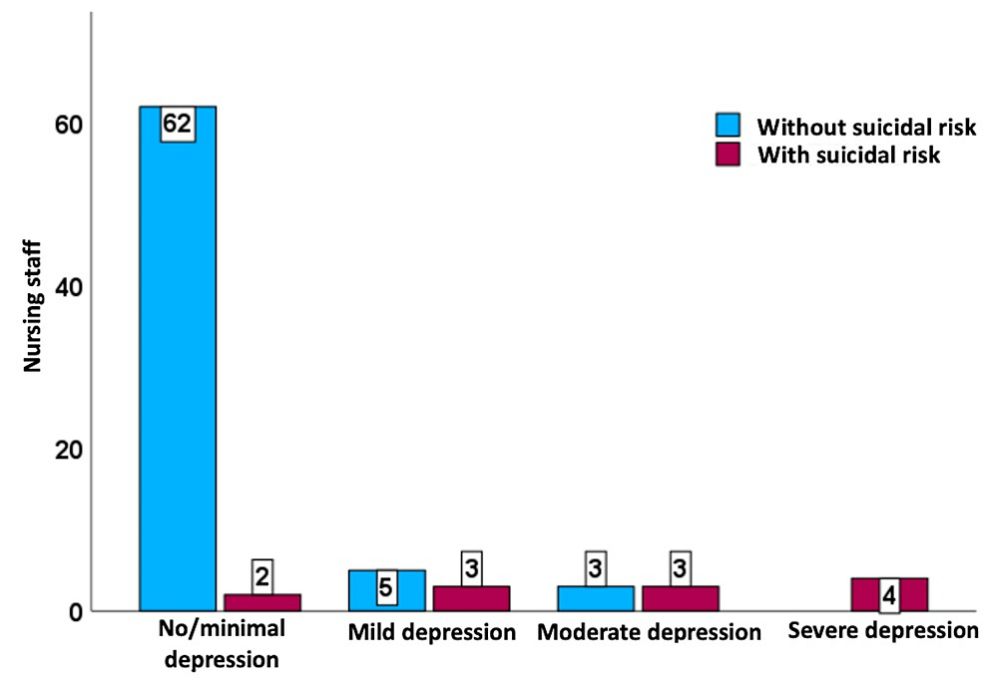
Distribution of the nursing staff (n=82) according to suicide risk and severity of depression in a Honduras National Hospital

Additionally, among the four participants experiencing severe depression, all four demonstrated a suicide risk; out of the six participants with moderate depression, three presented a suicide risk; among the eight participants with mild depression, three exhibited a suicide risk; and from the 64 participants displaying no or minimal depression, two had a suicide risk (Figure 3).

## Discussion

Our research revealed that the average age was 34.91 years, which aligns closely with a Mexican study, where general healthcare staff averaged 39.2 years [7]. Age group distribution showed 45.12% (n=37) in the 26-35 range, and 18.29% (n=15) in the 20-25 group, similar to a Brazilian study by de Vargas and Vieira in 2010, where 55% of their participants were aged 21-30. In our study, 51.2% reported being single, which was equal to the Brazilian study [8].

Regarding gender distribution, it was found that 86.59% of the nursing personnel were women, while 13.41% were men, a percentage similar to a study in Brazil by da Silva and colleagues in 2017, where 90.5% of the studied population were women, and the study by Groves et al. [9,10].

In terms of work area, 54.88% of the nursing staff worked in inpatient similar to a study in Mexico, where the majority were from the emergency department at 42.4% [11]. Only 15.9% of nursing staff engaged in employment across various institutions, mirroring findings from a 2018 Australian study that recorded a 9.8% proportion of nurses with multiple workplace commitments [12]. However, this stands in contrast to a Brazilian study where a significant 58% of participants held multiple work affiliations [8].

Elements that could impact the mental well-being of the study’s nursing staff include their involvement during the COVID-19 outbreak as 76.8% (n=63) worked during it. A recent investigation revealed that a quarter of healthcare professionals who worked in two nursing facilities during COVID-19 exhibited a heightened susceptibility to suicide [13].

In terms of tenure at the Mario Catarino Rivas National Hospital, the largest proportion (31.71%) had one to five years of service, while 25.61% had less than one year, differing from Lerma-Martínez et al.'s study where the highest percentage (23.7%) had 6-10 years of service, followed by 22% within the one to five year range [11].

Regarding daily work hours, 67.07% of nursing staff worked eight hours per day, followed by 9.76% who worked 10 hours per day. Barcellos et al. mentioned a median of 42 weekly working hours in their study [14].

Concerning the severity of depression as assessed by the BDI-II, 21.95% exhibited a degree of depression. These results are compared to Abraham's 2021 findings, where the aggregated prevalence of depression among healthcare workers was 33%, slightly differing from this study's results [15]. Consistently, Lerma-Martinez et al. showed a 20.3% prevalence of depression, and Vargas and Vieira demonstrated a 30% prevalence of some degree of depression among healthcare personnel [8,11]. In contrast, an Egyptian study shows that 79% of their nursing staff exhibited depression [16].

With regard to age and severity of depression in the nursing staff of our study, young adults had the highest prevalence of all three levels of depression. The youth showed an equal distribution across all three depression levels. Intermediate adults and senior citizens only experienced mild depression. Adolescents had only moderate depression cases. Per the United States National Institute of Mental Health's 2021 data, major depression affected 18.6% of individuals aged 18-25, 9.3% of those aged 26-49, and 4.5% of those over 50 years old. While specific data for the nursing community wasn't available, the general population's figures align with our study, indicating a predominant occurrence of depression among young adults [17].

Based on their work area, the inpatient area had 9.8% of participants experiencing depression, while the emergency department also had 9.8%. Both areas also showed an increased risk of suicide. Notably, Lerma-Martínez et al. found high anxiety and depression rates among nurses responsible for acute patients in their study [11].

The current study found that 14.63% of the sample was at risk of suicide, which contrasts with Tomás et al.'s study, where 6.2% showed suicidal risk [18]. A recent study found that nurses were 18% more likely to die from suicide than the general population [19]. 

Out of the 12 nurses who presented suicide risk, young adults were the most at-risk age group, followed by the youth. The majority of those at risk are associated with the emergency department, indicating a potential stressor or factor specific to that department that might be contributing to the risk. Half of the at-risk individuals worked in the inpatient department, slightly less than in the emergency department. All four participants with severe depression demonstrated a suicide risk. Half of the individuals with moderate depression in this group were at risk of suicide. It's noteworthy that even among those with no or minimal depression, there was still a small percentage that presented a suicide risk. A recent meta-analysis indicated that a diagnosis of major depressive disorder was linked to higher chances of ideation and mortality [20].

While this study aims to shed light on a relatively underexplored domain, it is important to take into account a number of limitations that should be considered in the interpretation of its results. The study's single-site approach and limited sample size of 82 nursing personnel from a specific hospital in San Pedro Sula might restrict the generalizability of the results to broader healthcare settings. The cross-sectional design captures a snapshot of data at a single point in time, preventing the establishment of causal relationships or the tracking of changes over time. The reliance on self-reported data introduces the potential for recall and social desirability biases. While the study identifies work areas with higher depression and suicide risk, it doesn't extensively explore the specific factors underlying these variations, nor does it deeply consider contextual, cultural, or external influences. 

## Conclusions

Our research provides a detailed examination of the demographics, work environment, and mental health of nursing staff. Even though the majority of the participants in our study presented no or minimal depression and no suicidal risk, the underlying prevalence of these mental health conditions emphasized the importance of early identification in all age groups and work areas of the nursing staff. However, this study is not without limitations, such as the focus on a single hospital, which may limit the generalizability of the findings. Future research should aim to include a more diverse range of healthcare settings and employ longitudinal designs to better understand the temporal relationships between work stressors and mental health outcomes among nursing staff.

Work area-specific mental health findings, particularly the elevated depression and suicide risk in the inpatient and emergency departments, underscore the multifaceted challenges faced by nursing professionals. The pronounced prevalence of depression and suicide risk calls for targeted interventions and robust support mechanisms to ensure the well-being of this critical workforce.
